# Adverse Side Effects: Empagliflozin-Related Acute Pancreatitis Case Report

**DOI:** 10.7759/cureus.12325

**Published:** 2020-12-27

**Authors:** Bassem S Zeidan, Charles Boadu, Andrea Hernandez, Johnathan Frunzi, Itioye Adetula

**Affiliations:** 1 Internal Medicine, HCA Medical Center of Trinity, Trinity, USA; 2 Internal Medicine, Medical Center of Trinity, Trinity, USA

**Keywords:** drug-related side effects and adverse reactions, types 2 diabetes, a sodium-glucose cotransporter 2 inhibitor, acute pancreatitis, abdominal pain, medications, side effects, adverse effects, empagliflozin

## Abstract

Acute pancreatitis is an acute inflammatory process of the pancreas that is associated with multiple etiologies. The two most common causes are gallstones and acute alcohol intoxication. However, medications are often overlooked when determining the cause. Empagliflozin is a type of sodium-glucose transport protein 2 (SGLT-2) inhibitor used for the treatment of type 2 diabetes mellitus. Given that this medication is new, the adverse effects have not been fully reported in the literature. Currently, the most commonly reported side effects are genitourinary infections such as cystitis or yeast infection although acute pancreatitis as a result of empagliflozin is very rare. Here, we discuss a case of a 64-year-old female who presented with severe pancreatitis after recently initiating the use of empagliflozin. Based on the timing of her presentation and her hospital workup to rule out many of the common etiologies, it was concluded that empagliflozin was the likely cause of her acute pancreatitis. With SGLT-2 inhibitors such as empagliflozin, becoming popular as first-line in the management of diabetes, this case may hope to raise awareness of the possible adverse effects related to it. Additionally, this case also emphasizes the importance of identifying iatrogenic related pancreatitis.

## Introduction

Acute pancreatitis is an acute inflammatory process of the pancreas. The two most common causes are gallstones and acute alcohol intoxication. Other etiologies include trauma, steroids, medications, scorpion stings, spider bites, malignancies, infections such as mumps, and autoimmune conditions. Sodium-glucose transport protein 2 (SGLT-2) inhibitors are the newest class of oral anti-hyperglycemic agents that have been approved for the treatment of diabetes mellitus [1]. The mechanism of action of these medications works in lowering blood glucose levels independent of insulin by binding to the SGLT-2 expressed in the proximal convoluted tubule of the kidney thereby limiting glucose reabsorption and blood glucose. Currently, there are three SGLT-2 selective inhibitors approved by the Food and Drug Administration (FDA) for mono, dual, and triple therapy: canagliflozin, dapagliflozin and empagliflozin [2]. Empagliflozin has the greatest selectivity for SGLT-2 compared to SGLT-1, while canagliflozin is the least selective [3]. Given how new these medications are, their adverse effects have to be fully reported in the literature. The most commonly reported adverse effect is glucosuria which consequently leads to genital-urinary infections such cystitis or yeast infection and osmotic diuresis [1]. Here we report a case of acute pancreatitis related to recent use of empaglifozin.

## Case presentation

**Figure 1:**
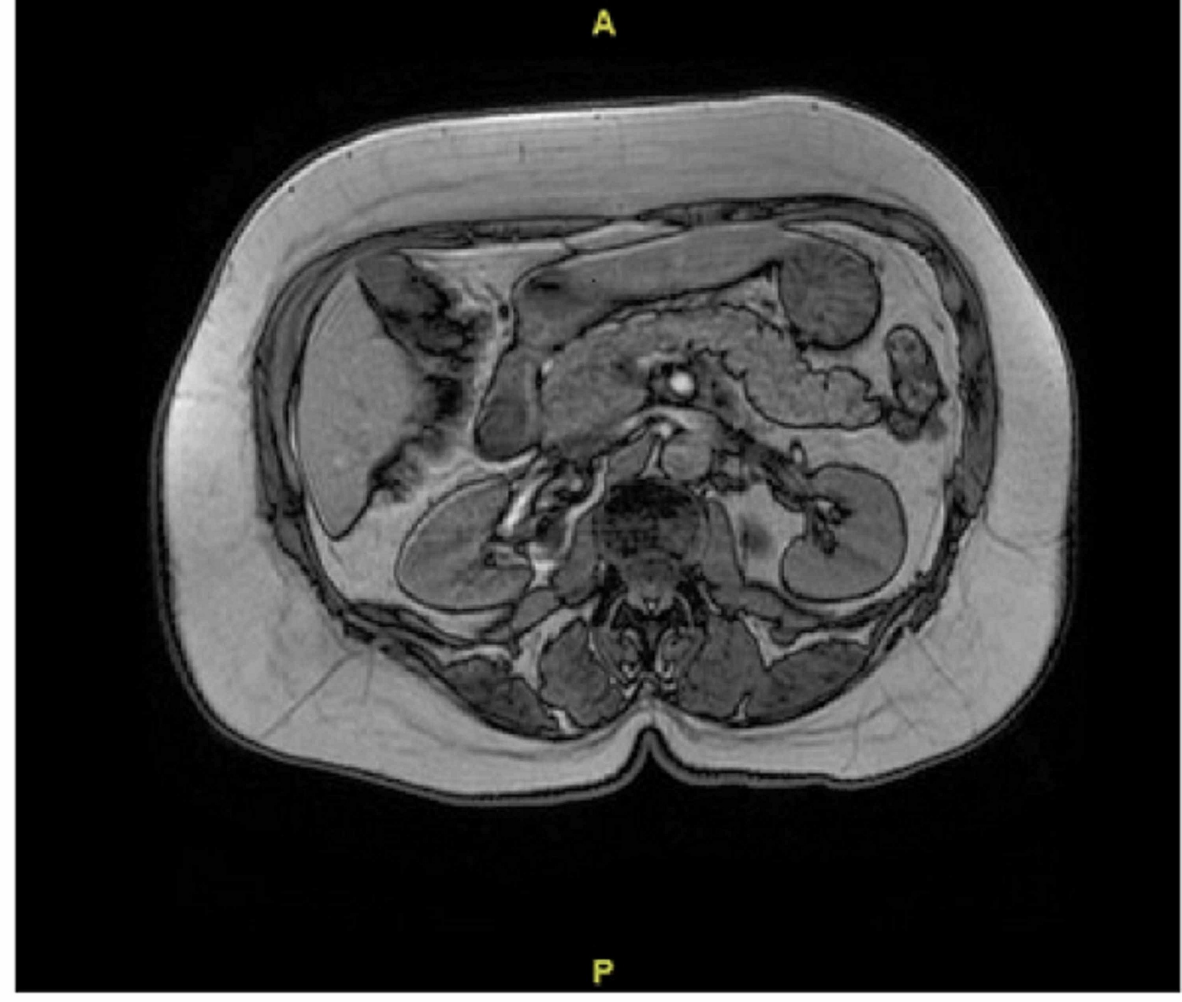
Magnetic resonance imaging/magnetic resonance cholangiopancreatography (MRI/MRCP): reporting mild enhancement and edema around the head and uncinate process of the pancreas and around the body and tail suggestive of pancreatitis.

We present the case of a 64-year-old Afro-Caribbean female with a past medical history of hypertension, hyperlipidemia, and type 2 diabetes admitted to the hospital for intractable abdominal pain that started six days prior to her presentation. It was located in the mid-epigastric region and radiated to the back, It was also associated with nausea and vomiting which worsened after eating. Her pain was also exacerbated with position changes such as leaning forward and was only alleviated by intravenous (IV) ketorolac given in the emergency department (ED). The patient denied any recent alcohol use, bug bite exposure or recent sick contacts. A review of her medication list included amlodipine 2.5 mg daily, nebivolol for 5 mg daily for years, and more recently, empagliflozin 10 mg daily which was started two weeks prior to symptoms onset. Family history was significant for breast, colon, and pancreatic cancers in first-degree relatives. On arrival, vital signs were stable. Physical examination, however, was significant for tenderness to palpation over the mid-epigastric region and right upper quadrant. ED laboratory workup was remarkable for elevated pancreatic lipase enzyme level 2110 units/L. A computed tomography (CT) scan of the abdomen confirmed mild pancreatitis involving the head and uncinate process, with adjacent edema and prominent lymph nodes (Figure 1).

**Figure 2:**
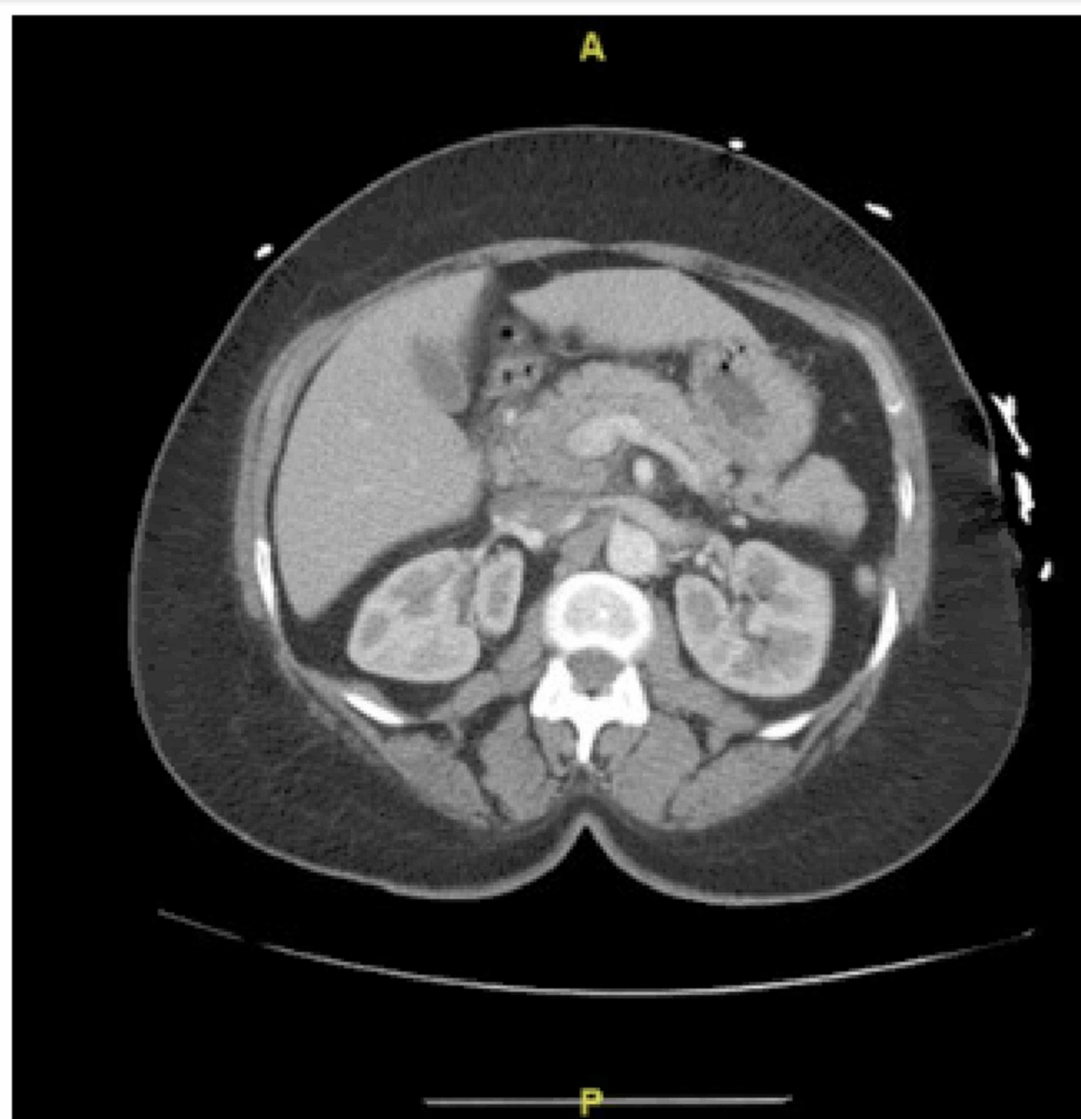
CT scan of the abdomen: mild pancreatitis involving the head and uncinate process, with adjacent edema and prominent lymph nodes.

The patient was admitted with the diagnosis of acute pancreatitis and underwent conservative management which included being made nothing by mouth, IV fluid resuscitation and underwent further studies including laboratory and advanced imaging to determine the etiology. Laboratory investigations involved a fasting lipid panel, and triglyceride level which were both within normal limits, and hemoglobin A1c (HbA1C) of 7.9%. Her initial workup also included an ultrasound of the right upper quadrant which was normal therefore ruling out any gallstones. Due to her significant family history and ominous presentation, we performed an abdominal magnetic resonance cholangiopancreatography (MRCP). This reported mild enhancement and edema around the pancreatic head and uncinate process and around the body and tail suggestive of pancreatitis (Figure 2). While on admission, her lipase levels returned to normal values and the patient resumed a normal diet. The empagliflozin was discontinued and she was started on insulin during her hospital course.

## Discussion

Acute pancreatitis continues to be a serious illness. Most patients are at risk to develop different complications from ongoing pancreatic inflammation [4]. It is therefore important to determine the underlying etiology to prevent further injury, recurrence, and complications. The two most common causes of acute pancreatitis are gallstones and acute alcohol intoxication. Other causes include trauma, surgery, infections, metabolic disorders, and medications. Drug-induced pancreatitis is rarely chronic and may be mild to catastrophic in severity, in the most severe cases leading to death [5]. Some of the common medications associated with drug-induced pancreatitis include angiotensin-converting enzyme inhibitors, statins, hormone replacement therapy, diuretics, HIV therapy, other anti-glycemic agents such as biguanides, dipeptidyl peptidase 4 (DPP4) inhibitors, and glucagon-like peptide-1 (GLP-1) mimetics [6]. Currently, there is a single case report that highlights the possible effects of empagliflozin-related pancreatitis [7]. However, the patient was also taking furosemide and metformin which are well known to be associated with pancreatitis [7].

What makes our case unique, is that the patient was chronically taking low dose amlodipine and nebivolol daily for hypertension and did not experience any side effects. While amlodipine is seldom known to cause acute pancreatitis, our patient had been taking a low dose of this medication daily for a number of years without any incident. Additionally, we did not come across any case reports in the literature of nebivolol-induced acute pancreatitis.

In our patient, the symptoms only developed after recently beginning empagliflozin as monotherapy for diabetes mellitus. During her admission, we continued her home blood pressure medications and empagliflozin was discontinued. The patient was started on low dose sliding scale insulin to manage her diabetes and symptoms improved within three days. Upon discharge, lipase levels had normalized and she tolerated a solid diet. In view of her extensive, yet unrevealing evaluation, we determined her acute pancreatitis was secondary to the empagliflozin and recommended the patient discontinue the medication. Upon discharge, she was placed on glyburide 2.5 mg daily and lisinopril 5 mg daily. We also recommended she continue her home amlodipine and nebivolol. She tolerated this new regimen very well and no longer experienced any abdominal pain per further chart review.

## Conclusions

SGLT-2 inhibitors are becoming more popular as first-line medications in the management of diabetes. This case demonstrates possible complications if clinicians opt to use these medications as monotherapy. Furthermore, this case highlights the importance of ruling out other etiologies of acute pancreatitis in order to determine if it is truly drug-related. Any drugs with the potential to cause acute pancreatitis should be discontinued or exchanged for a drug of a different class. If acute pancreatitis resolves after discontinuation of empagliflozin as in our patient, suspicion for drug-induced pancreatitis increases. The initiation of empagliflozin two weeks prior to the onset of her symptoms strengthens this relationship.
